# Spiritual Intelligence, Resiliency, and Withdrawal Time in Clients of Methadone Maintenance Treatment

**DOI:** 10.5812/ijhrba.11308

**Published:** 2013-12-22

**Authors:** Behnaz Shahbakhsh, Sedighe Moallemi

**Affiliations:** 1 Department of Psychology, Baharan Psychiatric Hospital, Zahedan University of Medical Sciences, Zahedan, IR Iran; 2 Deputy of Research, Zahedan University of Medical Sciences, Zahedan, IR Iran

**Keywords:** Intelligence, Substance-Related Disorders, Recurrence

## Abstract

**Background:**

Reports show an increasing interest in spirituality. It has been revealed that people with spiritual tendencies, can better deal with a trauma, manage the stressful situations, and have greater improvement in their health condition.

**Objectives:**

Our aim was to examine the relationship between spiritual intelligence and resiliency, and the relation of these two variables with the withdrawal time of individuals treated with methadone.

**Materials and Methods:**

This research was conducted on patients referred to the addiction center of Baharan Psychiatric Hospital in Zahedan, Iran. Our sample included 100 referrals; they were provided with questionnaires and asked to answer them honestly. King’s spiritual intelligence questionnaire and resilience questionnaires were used.

**Results:**

There were significant positive correlations between resiliency and scores of spiritual intelligence as well as with subscales of spiritual intelligence. In addition, there were significant positive correlations between withdrawal time and scores of spiritual intelligence as well as with subscales of spiritual intelligence as well as with resiliency.

**Conclusions:**

Relationships between the spiritual intelligence and resiliency parameters with withdrawal time show that these parameters can have a role in relapse protection among addicted people.

## 1. Background

Today more than ever before, people are witnessing stressful experiences that threaten their mental health, and are tended to maximize mental disorders in the suffering people. Living in an industrial world where humanity and social supports are gradually diminishing, proper human relationships are endangered. On the other hand, people being forced to make ceaseless efforts and ignore their wishes and innate needs, has brought about a harmful situation for mental health and wellbeing of the individuals (1).

The world health organization definition of health is not merely the absence of illness, but also the integration and coordination of welfare between physical, emotional, social and spiritual factors (2).

Reports show an increasing interest in spirituality. For example, Kantrovics et al. reported that 58 percent of Americans are seeking spiritual growth (1). Piedmont defines spirituality as an inner core self that can bring comfort to people particularly in stressful conditions and connect them to a superior force in the universe. Several researchers have shown that people with spiritual tendencies, when facing trauma, are better at dealing with their condition, managing stressful situations, and have greater improvement in their health condition (3-6). Emmons, by observing the behaviors and expressions of spiritual people such as Mother Teresa and Gandi, combined the spirituality and intelligence within a new structure and introduced spiritual intelligence to the word (7).

An individual with a high spiritual intelligence has flexibility, consciousness, the capacity for inspiration and intuition, and a holistic view of the world; he/she looks for an answer to fundamental questions of life and criticizes the traditions. Spiritual intelligence can easily change people. It is believed that different modes of resiliency are obtained during the process of failure and recoherence. The recoherence sustainer needs energy to grow; and it seems this energy has a spiritual and inherent source.

Seemingly, spiritual intelligence independently affects the mental health of individuals (8, 9). It is also possible that with its impact on resiliency, spiritual intelligence not only uses its positive effects to avoid problems, but also plays a vital role in problem solving and further, improves and expands the duration of withdrawal.

## 2. Objectives

The aim of this study was to examine the relationship between spiritual intelligence and resiliency, and the relation of these two variables with the withdrawal time of individuals treated with methadone.

## 3. Materials and Methods

This research was conducted on patients referred to the addiction center of Baharan Psychiatric Hospital in Zahedan, Iran. Our sample included 100 referrals, which were provided with questionnaires and asked to answer them honestly.

### 3.1. Tests Used

#### 3.1.1. King’s Spiritual Intelligence Questionnaire

The spiritual intelligence self-report questionnaire, developed by King DB (10), comprises of 24 items and 4 subscales including existential thinking, transcendental awareness, personal meaning production, and conscious state expansion, aiming to measure the spiritual intelligence. The scoring system is based on the Likert scale and has 4 subscales including as mentioned. High scores indicate high spiritual intelligence or the existence of such capacity.

Results of the exploratory factor analysis, conducted on 619 students of Trent University of Canada during 2007, showed that the Cronbach’s alpha coefficient was 0.95 and the obtained reliability from bisections was 0.84. Another study used confirmatory factor analysis, in which Cronbach’s alpha and standard alpha coefficients were both 0.92 (10).

In addition, in another study, Moallemi, Bakhshani and Raghibi (9) showed that the Cronbach’s alpha coefficient of the questionnaire was 89. Moreover, the reliability coefficient of the spiritual intelligence questionnaire, evaluated by test-retest in a sample of 70 individuals during 2 weeks, was 0.67.

#### 3.1.2. Resilience Questionnaire

Connor and Davidson developed the resilience questionnaire using the research resources from 1979 to 1991 (11). Psychometric properties of the scale were conducted on six groups including general population, patients referred to the primary care, psychiatric outpatients, patients with generalized anxiety disorders, and two groups of patients with post-traumatic stress (PTSD). The scale developers believed that this questionnaire is well able to distinguish the resilient and delicate personalities in clinical and nonclinical groups and can be used in clinical situations and researches.

Cronbach’s alpha coefficient was used as a measurement tool to calculate reliability. The reliability coefficient was 89% versus 87% for the factor analysis method. Besides undergoing initial standardization by Nikouzadeh, reliability of the questionnaire was calculated once more with the total alpha coefficient being 90% (12).

### 3.2. Methods

The participants were selected from patients referred to the addiction center of Baharan psychiatric hospital in Zahedan City. The questionnaires were given to those who were willing to cooperate and were in a relatively good physical condition. They were asked to honestly answer the questions related to their withdrawal time, demographic characteristics and other matters. The available method of sampling was used and everyone referred to methadone maintenance treatment (MMT) center was asked to complete the questionnaires during 2 months. We performed a correlation-descriptive research and chose those participants who hadn’t used any substance with the exception of methadone within the past 1 month.

### 3.3. Data Analysis

The analysis methods used in this research were descriptive statistic and Pearson correlation.

## 4. Results

As Table 1 shows, mean age of participants was 33.34 years with the youngest being 21 and the oldest 62, mean withdrawal time was 328 days with minimum of 38 and maximum of 1440 days, 64.4% were males and 35.4 % were females, 27.8% single and 67% married, and the most used substance was opium (74.4%). 

**Table 1.** Demographic Characteristics of Participants

Variables	Amount
**Age, y**	
Mean	33.34
Minimum	21
Maximum	62
**Time of withdraw, d**	
Mean	328
Minimum	38
Maximum	1440
**Sex, No. (%)**	
Male	51 (64.6)
Female	28 (35.4)
**Marital status, No. (%)**	
Single	22 (27.8)
Married	53 (67)
Miss	4 (5)
**Kind of used substances, %**	
Hashish	2.5
Opium	74.4
Crystal	21.5
Others	2.5

As shown by Table 2, there was a relationship between variables. Coefficient of correlation between resiliency and total score of spiritual intelligence were 0.44, and with the following subscales of spiritual intelligence: critical existential thinking (0.53), transcendental awareness (0.43), conscious state expansion (0.23), and personal meaning production (0.27). 

**Table 2.** Coefficients of Correlations Between Variables

	Spiritual Intelligence	Resiliency	Withdrawal Time	CET^a^	PMP^a^	TA^a^
**Resiliency**						
Pearson correlation	0.444^b^					
Sig.^a^ (2-Tailed)	0.000					
No.	66					
**Withdrawal time **						
Pearson correlation	0.263^c^	0.233^c^				
Sig. (2-Tailed)	0.030	0.045				
No.	68	74				
**CET**						
Pearson correlation	0.834^b^	0.530^b^	0.390^b^			
Sig. (2-Tailed)	0.000	0.000	0.000			
No.	68	74	76			
**PMP**						
Pearson correlation	0.578^b^	0.274^c^	0.054	0.403^b^		
Sig. (2-Tailed)	0.000	0.020	0.644	0.000		
No.	68	72	76	74		
**TA**						
Pearson correlation	0.635^b^	0.435^b^	0.265^c^	0.653^b^	0.139	
Sig. (2-Tailed)	0.000	0.000	0.020	0.000	0.238	
No.	68	72	77	74	74	
**CSE** ^**a**^						
Pearson correlation	0.724^b^	0.231^c^	0.251^c^	0.495^b^	0.311^b^	0.310^b^
Sig. (2-Tailed)	0.000	0.048	0.026	0.000	0.006	0.006
No.	68	74	79	76	76	77

^a^ Abbreviations: CET, critical existential thinking; CSE, conscious state expansion; PMP, personal meaning production; TA, transcendental awareness; Sig, Significant.

^b^ Correlation is significant at the 0.01 level (2-tailed).

^c^ Correlation is significant at the 0.05 level (2-tailed).

Another variable was withdrawal time which had a correlation with spiritual intelligence (0.26), critical existential thinking (0.39), transcendental awareness (0.26), conscious state expansion (0.25), and resiliency (0.23).

## 5. Discussion

Based on the results of this study, the first hypothesis related to the relationship between spiritual intelligence and resiliency was supported by the results. There was a positive and significant correlation between spiritual intelligence and resiliency. Furthermore, there was a significant positive correlation between resiliency and other subscales of spiritual intelligence. Hamid et al. who performed a research on spiritual intelligence and resiliency, reported a significant positive correlation between resiliency and spiritual intelligence (8).

A recent study showed that there was a significant relationship between the scores of spiritual intelligence and resiliency. The study was performed on a group of nurses who showed that having a meaning and purpose in life as well as a set of strong beliefs and views are their work requirements, which was in accordance with the characteristics of spiritual intelligence. Perhaps the way people feel about supernatural phenomena can provide them with psychological and spiritual supports which cannot be measured in terms of phenomenology (13). In the view of Vagan, spiritual intelligence slicks together all human aspects and can change and manage our lives (14). Due to the nature of transcendent spiritual experiences, people with such beliefs continuously have a sense of spiritual and divine interventions, which can change their life events as well as their thoughts and behaviors, and can help them cope with unpleasant events.

Moreover, when people need assistance to deal with life pressures, the spiritual realm can help them find meaning in stressful situations and through that, they can more easily cope with the circumstances (13). The correlation between subscales and resiliency is close to the correlation coefficient reported by Hamid et al. and Akbarzadeh et al. (8, 13). Other researches (3-6) have indicated that people with spiritual tendencies can confront the difficulties in a better way, manage the stressful situations, and have greater improvement in their health condition.

Emmons, by observing the behaviors and manners of spiritual personalities such as Mother Teresa and Gandi, combined the structures of spirituality and intelligence to form a new structure and introduced the term “spiritual intelligence” (7). After that, researchers tried to measure the relationship between spiritual intelligence and variables such as resiliency.

The word “resiliency” was first introduced by Suzanne Kobosa and Salvatory Maddey (15). They and other researchers (16-18) showed that people with high resiliency don’t get sick in stressful situations. These people are deeply involved in life activities and overcome the challenges of life. They believe that they are not a passive audience and on the contrary they live a purposeful life and accept responsibilities in order to make their life meaningful. Committed individuals tend to interpret life events as meaningful experiences and believe that all life activities have a general objective (19).

Other hypothesis was related to the significant relationship of spiritual intelligence and resiliency with withdrawal time, the correlations of which were supported by our results. Withdrawal time correlated with spiritual intelligence, and other subscales except personal meaning production and resiliency, were positively related to withdrawal time. Furthermore, due to lack of rich literatures on the impact and role of spiritual intelligence and resiliency on withdrawal time , more researches in this sphere are strongly suggested.
